# Trifascicular Block and Ventricular Standstill: A Late Complication of Mediastinal Radiotherapy in a Cancer Survivor

**DOI:** 10.7759/cureus.12806

**Published:** 2021-01-20

**Authors:** Sadaf Ali, Omer Ali, Irfan Ahmed, Tahir Nazir

**Affiliations:** 1 Internal Medicine, Lancashire Teaching Hospitals NHS Foundation Trust, Royal Preston Hospital, Preston, GBR; 2 Cardiology, Lancashire Teaching Hospitals NHS Foundation Trust, Royal Preston Hospital, Preston, GBR

**Keywords:** cardiac rhythm abnormalities, syncope, radiotherapy

## Abstract

Over the last half-century, radiation therapy has evolved to become one of the cornerstones of treatment for various types of cancers. It is estimated that more than 50% of patients with cancer are treated with radiotherapy. Patients with early stages of some cancers can even achieve a cure with radiotherapy alone. Radiation-induced heart disease is a well-recognized cause of mortality and morbidity in cancer survivors as a late complication of radiotherapy, often occurring more than a decade after radiotherapy. We describe a case of a middle-aged female who presented to the hospital with syncopal episodes. She was in remission from non-Hodgkin's lymphoma having received mediastinal radiotherapy 20 years, previously. Her initial workup such as laboratory investigations and 12 lead electrocardiogram were largely unremarkable. Cardiac monitoring over the course of the next few days was consistent with complete heart block with evidence of ventricular standstill. Her symptoms resolved following the implantation of a dual-chamber cardiac pacemaker. This case highlights the significance of clinical history taking and putting together all relevant facts to come to a differential diagnosis. In our case, this could have been easily overlooked as radiation therapy was given many years previously. We review and present an up-to-date albeit brief literature review on long-term cardiovascular complications of radiotherapy. Radiation-induced cardiac complications are an important cause of mortality and morbidity in cancer survivors. This article aims to raise awareness amongst clinicians of cardiac adverse effects occurring several years after the radiation therapy. This case also highlights the need for further research to better understand the pathophysiology of cardiovascular disease post-radiotherapy in order to develop effective prevention strategies and improve clinical outcomes.

## Introduction

Radiotherapy constitutes an important treatment modality for cancer and its use has led to improved survival in patients with many types of malignancies. Subtle sub-clinical cardiac alterations resulting from radiotherapy increase the risk of overt radiation-induced heart disease decades after radiotherapy [1]. This could be of particular significance in younger patients having a malignant disease with excellent prognosis such as breast cancer, Hodgkin’s, and non-Hodgkin’s lymphoma. The number of long-term cancer survivors after radiotherapy is increasing due to the concurrent use of newer targeted therapies including checkpoint inhibitors. Therefore, an improved understanding of radiation-induced heart disease is becoming increasingly important and clinically relevant [2]. Radiation-induced heart damage includes acute cardiac inflammation at the time of radiotherapy or shortly afterward, causing myocarditis or pericarditis. Late cardiovascular effects can clinically manifest themselves, decades after the index treatment resulting in various complications such as myocardial fibrosis leading to systolic or diastolic heart failure, valvular heart disease, vasculopathy including coronary artery disease, and conduction system dysfunction manifested as arrhythmias or various degrees of heart blocks. It is estimated that up to 75% of long-term cancer survivors receiving mediastinal radiotherapy may have conduction defects on routine electrocardiogram [3].

## Case presentation

We report a case of a 53-year-old woman who presented to the emergency department with dizzy spells and episodes of syncope. Her past medical history included Hodgkin's lymphoma for which she had received mediastinal radiotherapy more than 20 years previously and was in disease remission. She described having several episodes of transient loss of consciousness over the last six months, each lasting for 20 seconds. There were no accompanying symptoms of palpitations, chest pain, shortness of breath, or diaphoresis. Previously, she was referred to a neurology clinic for these symptoms and a subsequent computerized tomography scan of the brain and electroencephalography (EEG) were both found to be within the normal range. Hence neurologist referred her to the cardiology team for an opinion.

**Figure 1:**
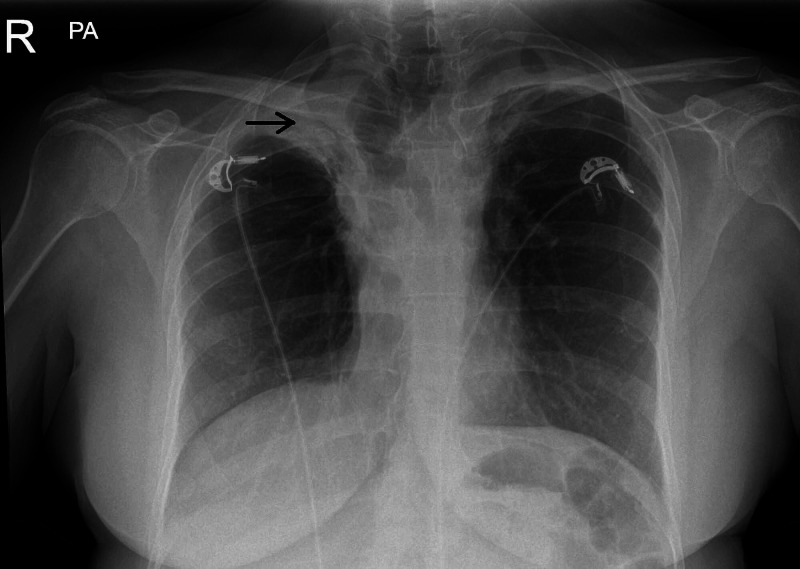
Chest X-ray film showing extensive volume loss in both upper lobes, more marked on the right side and tracheal deviation to the right, secondary to previous mediastinal radiotherapy (arrow)

Whilst she was waiting to be seen in cardiology outpatients, she was admitted to the hospital again with an episode of dizziness and loss of consciousness via the emergency department. On initial assessment, her bedside observations were normal, with a heart rate of 80 beats per minute and blood pressure of 130/80 mmHg, and no evidence of postural drop. Systemic clinical examination was unremarkable with normal heart sounds and clear chest on auscultation. Detailed neurological examination was normal. Laboratory investigations including full blood count, renal functions, electrolytes, thyroid function tests, serum glucose, serum cortisol, and cardiac enzymes were all within the normal range. Plain chest X-ray film showed normal heart size with extensive volume loss in both upper lobes, more marked on the right side and tracheal deviation to the right, secondary to previous mediastinal radiotherapy (Figure 1).

**Figure 2:**
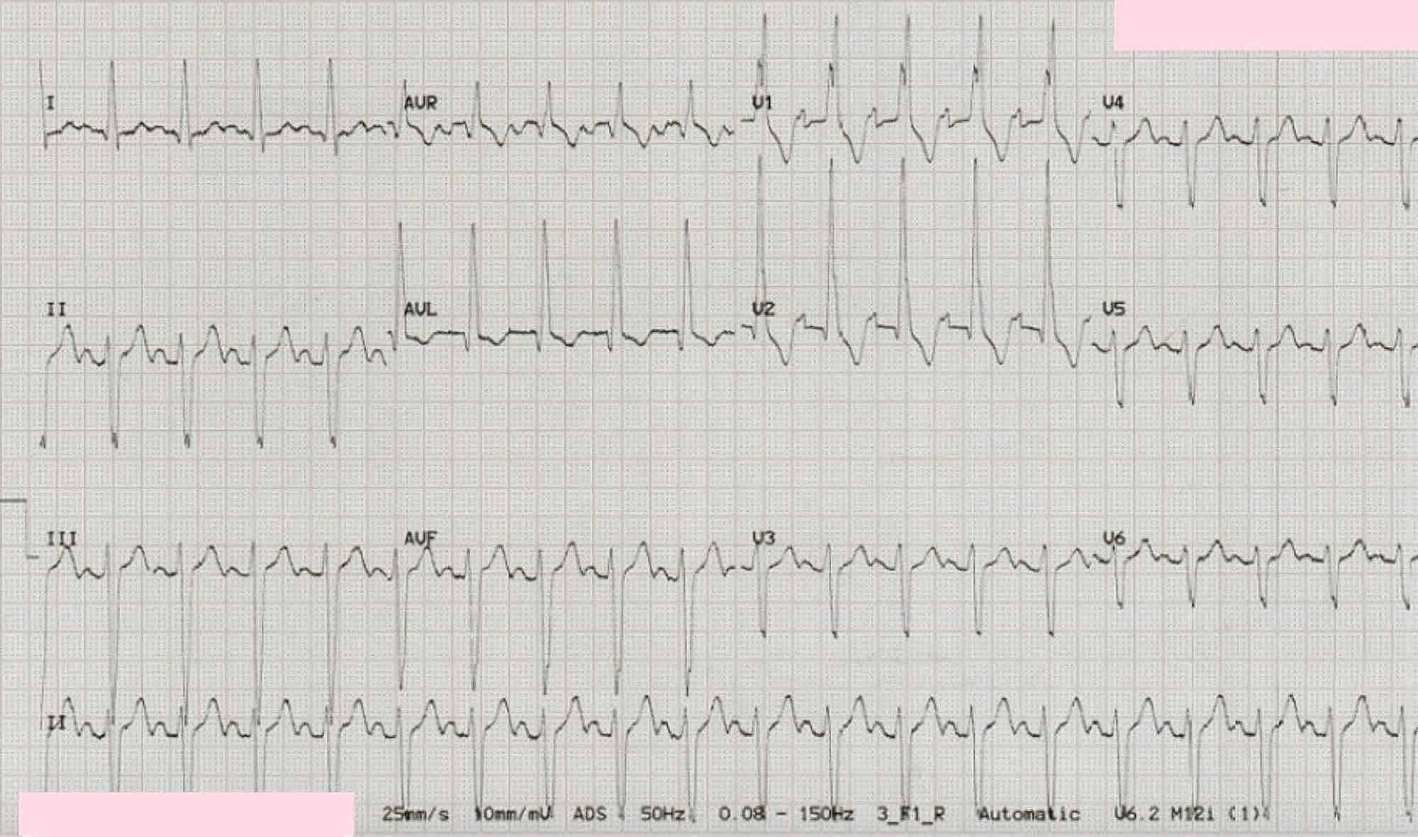
A 12 lead electrocardiograph (ECG) showing sinus rhythm with a ventricular rate of 110/min, first-degree heart block, right bundle branch block, and left anterior hemiblock in keeping with trifascicular heart block

A 12 lead electrocardiograph (ECG) showed sinus rhythm with a ventricular rate of 110/min, first-degree heart block, right bundle branch block, and left anterior hemiblock in keeping with trifascicular heart block (Figure 2).

Transthoracic echocardiogram showed normal left ventricular dimensions and function. There were no significant valvular abnormalities

**Figure 3:**
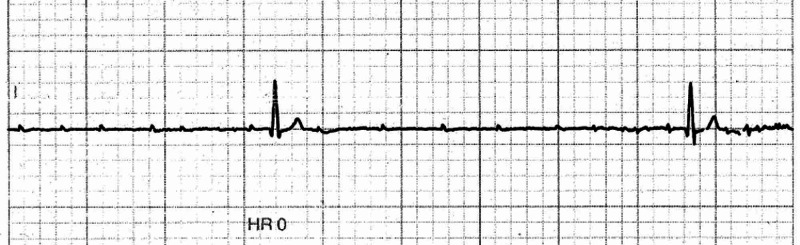
Cardiac rhythm strip showing ventricular standstill with a five seconds pause with only P waves visible

Due to her presenting complaint and ECG findings, she was admitted to the coronary care unit, and cardiac monitoring was initiated. Within 24 hours of admission, she had another episode of syncope while lying in the bed. Cardiac monitoring during this episode of transient loss of consciousness showed ventricular standstill for five seconds with only P waves visible (Figure 3).

**Figure 4:**
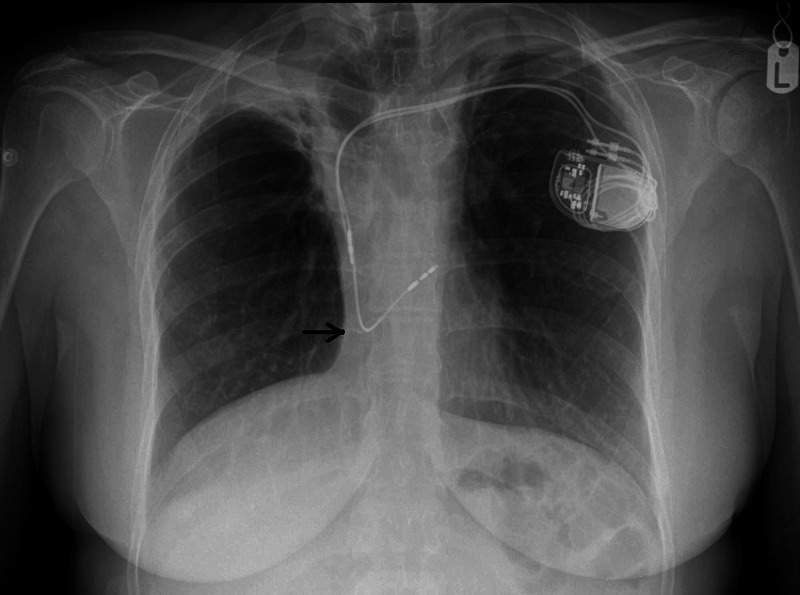
Post permanent pacemaker implantation, chest X-ray shows active leads fixed to the right atrial appendage and right ventricular apex (arrow)

A dual-chamber permanent pacemaker device was implanted with the active leads fixed to the right atrial appendage and right ventricular apex (Figure 4).

This led to a complete resolution of her symptoms. She made an excellent recovery post-procedure and episodes of dizziness and syncope resolved.

## Discussion

Radiotherapy is a well-recognized and established treatment for malignancies. It forms an integral component of therapeutic strategy with a view to either achieve a long-term remission or disease control in a number of intrathoracic malignancies including Hodgkin’s lymphoma, breast cancer, lung, oesophagus, and thyroid cancer [4]. It can be delivered through the external beam, internal placement or systemic administration, depending on the type of cancer and treatment goals, with an aim to target cancerous cells [5].

Undoubtedly, cancer survival has improved in the last couple of decades due to therapeutic advances and early diagnosis of the disease. However, the treatments for malignancies such as radiotherapy and chemotherapy result in both short-term and long-term adverse effects. These complications can affect any organ system and often lead to clinically significant sequelae due to the involvement of cardiovascular, pulmonary, and gastrointestinal systems. Radiation-induced tissue damage resulting in pneumonitis, mucositis, esophagitis, enteritis, proctitis, and cystitis is well recognized and routinely encountered in clinical practice [5].

Cardiovascular complications of cancer and its treatment lead to significant morbidity and mortality in cancer survivors [5]. In particular, cardiovascular adverse effects of radiotherapy can clinically manifest themselves, several years or even decades after the initial treatment. These complications include myocardial fibrosis, valvular heart disease, coronary artery disease, pericardial disease, and conduction system dysfunction [3]. Whilst the exact pathophysiological mechanisms underpinning these complications are not fully understood, a plausible theory suggests that radiations cause damage to cardiomyocytes, and as they lack the ability to replicate; these effects can last long-term. Support cells such as fibroblasts may proliferate in an attempt to limit the damage and often result in tissue fibrosis. However, with different cells in the vasculature including endothelial cells, smooth muscle cells, and macrophages having the ability to replicate and renew themselves, it is difficult to comprehend how relatively short-term insults given by cancer treatments show significant vascular effects in the long-term [6]. Although the pathophysiology is complex, inflammation and reactive oxygen species both seem to play a key role in causing permanent DNA damage. With the growing number of patients receiving radiotherapy, further studies are needed to understand the pathophysiological mechanisms to risk stratify individuals at risk, plans for prevention and identify molecular targets for treatment [6].

Cardiac conduction system may also get involved in radiotherapy-induced tissue fibrosis although the true incidence of this is not known. Nevertheless, the presence of conduction abnormalities on 12 lead ECG signifies the extent of underlying cardiac tissue damage [7]. As many as three-quarters of long-term cancer survivors may have conduction defects on the routine ECG trace following mediastinal radiotherapy. These conduction abnormalities range from minor changes such as prolongation of PR interval and right bundle branch block on one hand to serious and life-threatening conditions like complete atrioventricular dissociation and sick sinus syndrome, on the other [7].

Due to the wide heterogeneity in clinical manifestations of cardiac conduction abnormalities, patients with cardiac symptoms post-radiotherapy require a diligent clinical assessment [8]. Patients with complete heart block may present with pre-syncope, syncope, palpitations, low blood pressure, or increased breathlessness [8]. A 12 lead ECG, cardiac monitoring, and echocardiogram are useful initial investigations that help in the diagnosis of conduction abnormalities and can also explore other cardiac manifestations of radiotherapy including valvular pathologies, myocardial fibrosis, and regional wall motion abnormalities.

The exact treatment of the radiotherapy-induced cardiac conduction abnormalities depends upon their precise nature and severity. Those with complete atrioventricular dissociation (as in our case) require a permanent pacemaker. However, if the complete heart block is accompanied by left ventricular systolic dysfunction, cardiac resynchronization therapy (CRT-P) may be preferable. Due to the nature of cardiovascular manifestations presenting decades after initial radiotherapy, these patients do warrant a life-long follow-up [9]. Those with pre-existing cardiovascular risk factors are at a higher risk and effort should be made to treat modifiable risk factors by using guideline-directed therapy. Preventative strategies such as alteration in the radiotherapy field or targeted radiation, with shielding of the heart, may help to minimize radiation-induced cardiac damage.

## Conclusions

Radiotherapy remains an essential treatment strategy for many cancers including those affecting the younger population. Serious cardiovascular complications may arise several years or decades after the initial radiation treatment. Clinicians must keep an index of suspicion when dealing with cancer survivors presenting with cardiovascular problems. With the ever-increasing risk of cancer with age and increased prevalence of cardiac disease in an older population, further work is needed to risk-stratify patients prior to commencing radiotherapy. Longer-term surveillance of young cancer patients with higher life expectancy is equally important.
